# Learning from the Past to Improve the Future—Vaccine Hesitancy Determinants in the Italian Population: A Systematic Review

**DOI:** 10.3390/vaccines11030630

**Published:** 2023-03-12

**Authors:** Michela Ferrara, Giuseppe Bertozzi, Gianpietro Volonnino, Aldo Di Fazio, Nicola Di Fazio, Mauro Arcangeli, Raffaele La Russa, Paola Frati

**Affiliations:** 1 Department of Anatomical, Histological, Forensic Medicine and Orthopedic Sciences, Sapienza University of Rome, 00185 Rome, Italy; 2 Department of Clinical and Experimental Medicine, Section of Forensic Pathology, University of Foggia, 71122 Foggia, Italy; 3 Regional Complex Intercompany Institute of Legal Medicine, 85100 Potenza, Italy; 4 Department of Clinical Medicine, Public Health, Life and Environment Science, University of L’Aquila, 67100 L’Aquila, Italy

**Keywords:** vaccine hesitancy, infodemic, COVID-19

## Abstract

WHO identifies vaccine hesitancy (VH) as one of the ten threats to global health. The authors bring to the international scientific community an Italian episode that offers the opportunity to renew the discussion on the extent of the VH matter. The purpose of this systematic review is to analyze the factors determining vaccine hesitancy in the Italian population, to understand its roots, and suggest potential strategies to mitigate it. A systematic review of the literature according to the PRISMA guidelines was carried out using the SCOPUS and Medline (via PubMed) databases, using the following strategy: (COVID-19 vaccines) AND (vaccination hesitancy) AND (Italy). After the selection process, 36 articles were included in this systematic review. The most frequently detected factors associated with VH in the Italian population can be grouped as vaccine-related factors, socio-cultural factors, and demographic factors. Currently, we are facing a gap between the population and science, governments, and institutions. To heal this breach, it is necessary to strengthen the trust of the population through the implementation of health communication and public education strategies, while scientific literacy must continue to support families and individuals in discerning evidence from opinions to recognize the real risks and balance them with the benefits.

## 1. Introduction

In the topical background characterized by the spread of the COVID-19 disease, vaccines represent the most effective tool in containing the pandemic.

According to official data from the World Health Organization (WHO), to date, over thirteen billion doses have been administered worldwide [1] and, although the safety of immunization has been widely demonstrated, there are still many people sceptic about vaccination due to misconceptions or distrust of the scientific evidence [2].

WHO defines vaccine hesitancy as “the delay in acceptance or refusal of vaccination” and identify it as one of the ten threats to global health [3]. This phenomenon depends on several factors, including socio-demographic, cultural, and religious beliefs [4,5,6,7,8], and is often fueled by political debates that sometimes provide unreliable information. An additional role is played by the influences of anti-vaccination movements and social media, which feed the so-called infodemic phenomenon, meaning the dissemination of false or misleading information not based on scientific evidence [9,10,11,12,13]. Frequently, the eventuality of vaccination-related side effects is overemphasized, leading to an attitude of suspicion and rejection from the population. This setting constitutes a danger both to public health and the individual in the pandemic era, sometimes resulting in significant consequences in therapeutic choices not always directly related to vaccination itself.

The authors want to bring to the international scientific community the Italian case of a married couple who asked to use the blood of people unvaccinated against the coronavirus for possible transfusions during the life-saving heart surgery of their two-year-old child, postponing the operation. The health workers, believing that they could no longer delay the surgery, challenged Article 3 of Law 219 of 2017, which provides that if the legal representatives of the minor refuse the treatment deemed appropriate and necessary by the doctor, the decision is left to the tutelary judge. The dissent of the couple derived from the assumption that the COVID-19 vaccine involves a high incidence of cardiovascular complications and can be inoculated through transfused blood. However, after analyzing the reasons given by the spouses, the tutelary judge emphasized in agreement with the scientific community that blood transfusion does not involve any risk for patients who receive blood from vaccinated subjects. Therefore, the judge suspended the parental authority of the couple and ordered that the child undergo cardiac surgery, with possible blood transfusions chosen by the hospital. The case is peculiar as it highlights a new aspect of the “dangerousness” of the distrust of the vaccine against COVID-19, whose perceived risk, based on non-scientifically founded beliefs, is such as to lead to the decision to delay a life-saving intervention.

The episode described above offers the opportunity to renew the discussion on the extent of the “vaccine hesitancy” matter. VH has a complex nature and varies according to time and country [14,15]. Therefore, although numerous studies investigating this phenomenon worldwide have been published in the literature, they may have the limitation of not investigating the determinants in relation to the context of a specific region. The purpose of this systematic review is to analyze the factors determining vaccine hesitancy in the Italian population, to understand its roots, and suggest potential strategies to mitigate it.

## 2. Materials and Methods

On 9 November 2022, a systematic review of the literature according to the preferred reporting items for systematic reviews and meta-analyses (PRISMA) guidelines [16] was carried out using the SCOPUS and Medline (via PubMed) databases, using the following strategy:

− Scopus search string: (COVID-19 vaccines) AND (vaccination hesitancy) AND (Italy);

the filters applied were Document type: Article, Short Survey; Language: English.

− Pubmed search string: ((“COVID-19 Vaccines”[Mesh]) AND “Vaccination Hesitancy”[Mesh]) AND “Italy”[Mesh];

The filter applied was Language: English.

### 2.1. Inclusion and Exclusion Criteria

The inclusion criteria were: human studies; studies among healthcare workers, pregnant women, parents, students, and people affected by pathologies; aim of the study: to evaluate COVID-19 vaccine hesitancy in the Italian population. The exclusion criteria were: article not aimed at evaluating COVID-19 vaccine hesitancy in the Italian population; article not in English; abstract; case report; editorial; review; letter; note; conference paper.

### 2.2. Quality Assessment and Critical Appraisal

M.F. and G.B. evaluated the entire text of the articles, independently. The articles in which there was disagreement were discussed with the senior investigator, P.F., for the final decision.

### 2.3. Risk of Bias

The main risk was linked to the keyword selected for the search strings. Therefore, the Kappa interobserver variability coefficient showed “almost perfect agreement” (0.89) [17].

### 2.4. Characteristics of Eligible Studies

**Figure 1:**
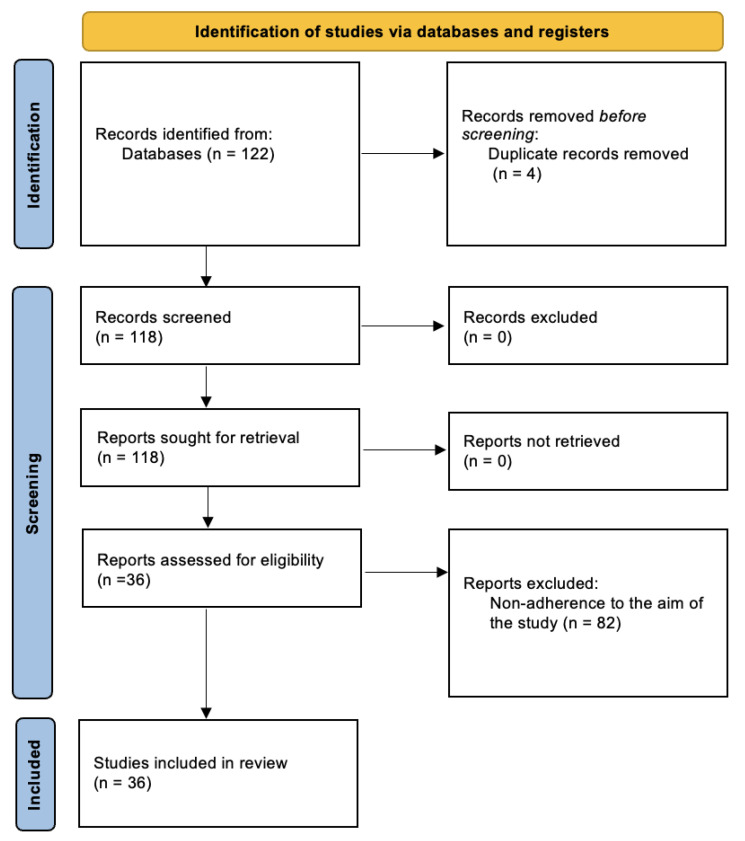
Prisma flow diagram for this systematic review.

A total of 122 articles were identified. Four duplicate articles were removed, and 82 articles did not meet the inclusion criteria. After the selection process, 36 articles were included in the present systematic review (Figure 1).

## 3. Results

The analysis of the results, summarized in Table 1, shows that the overall period covered by the included studies ranges from December 2020 to May 2022.

Of these, four studies were performed before the vaccination campaign in Italy (December 2020), 29 studies were conducted after that date, and three studies started before and ended after the vaccination campaign began in Italy. In relation to the type of population under study, the articles included in this review can be divided as follows: 10 studies investigated the phenomenon of vaccination hesitancy in pregnant women or parents/caregivers; 10 studies concern medical students or health professionals; six studies consider individuals affected by pathologies; and 10 studies analyze the problem in the general population (including non-healthcare students).

The most frequently detected factors associated with VH in the Italian population are summarized in Table 2.

These factors can be grouped into three main categories: vaccine-related factors (e.g., doubts about the safety and effectiveness of the vaccine, concern about adverse reactions, awareness of the possibility of contracting the infection, adequacy of information regarding the topic); socio-cultural factors (e.g., low level of education, political orientation, social support, distrust in authorities); and demographic factors (gender, age).

### 3.1. Vaccine-Related Factors

The most common predisposing factor for VH found in the included studies and related to vaccination is the fear of developing adverse reactions [19,23,24,25,29,34,37,38,39,43,45,49]. Another significant aspect is the distrust or doubts about vaccination against COVID-19 and its efficacy [20,21,24,27,34,38,44,49,51,52,53]. As easily expected, the attitude of refusal towards vaccinations or the adoption of poorly protective behaviours constitutes a further preponderant element predisposing to VH against COVID-19 [24,26,27,28,30,37,39,40,42,48,51]. Another predisposing element is the lack of awareness of contracting the infection [18,31,33,34,37,42,44,46,49]. Furthermore, four studies found that knowing people who have contracted the COVID-19 disease affects the decision to undergo the vaccination [26,30,33,42]. Finally, the preference for natural immunity or having contracted COVID-19 before vaccination are both conditioning factors for the decision to undergo the vaccine [24,35,38,39].

### 3.2. Socio-Cultural Factors

Among the sociocultural factors, the one most frequently found associated with VH is a low level of education [18,26,27,30,32,40,41,44,50,51,53]. Information obtained through mass media or the internet, scarcity of information, and information obtained from non-medical people are associated with VH [18,23,27,37,50,52]. Five studies identify distrust of authorities [22,28,30,37,48], and two studies identify a conspirative mentality [28,30] as elements implied in the decision to vaccinate against COVID-19. One study found that VH is widespread in individuals with right-wing or center-right political orientation [22], while another survey shows that the phenomenon is associated with poor social support from parents or family members [30].

### 3.3. Demographic Factors

Three studies considered age as a predisposing factor for VH. Tomietto et al. [19] show that those belonging to the so-called “generation X” (born between 1961 and 1980) [54] are more hesitant. Moscardino et al. [30], on the other hand, found that reluctant people fall more into the 30–40 age group. According to the study by Zona et al. [44], individuals younger than 40 are the most unwilling to vaccinate against COVID-19. In addition, one study reveals that the inhabitants of northern Italy and those who are unemployed are more hesitant towards this vaccine [30]. Five studies show that female gender is an additional determinant for hesitancy [27,40,50,51,53].

Analyzing the phenomenon in relation to the different categories of individuals taken into consideration in this review, it emerges that, as regards the population of “parents”, including pregnant women, the factor most frequently found as predisposing to vaccination hesitancy is a low level of education, encountered in 7 out of 10 studies. The fear of developing adverse reactions to vaccination is the factor most highlighted in the category of “health professionals and medical students” and in the population of people affected by pathologies, although there is no apparent prevalence of this element compared to the other determinants found in these individuals. On the other hand, studies conducted on the general population show that the main predisposing factors to vaccine hesitancy consist of distrust of the authorities and an attitude of general refusal towards vaccinations.

## 4. Discussion

Vaccine hesitancy is an ever-current topic and understanding its determinants may help both to develop strategies to enhance immunization acceptance and learn lessons from the pandemic to be better prepared for future public health crises. The data updated to 2 December 2022 show that in Italy, 6.79 million people over the age of 5 have yet to receive even one dose of the COVID-19 vaccine, of which 6.10 million are currently eligible for vaccination [55]. This review analyzes the factors predisposing to vaccine hesitancy in the Italian population and represents, to the best of our knowledge, the first systematic review providing a global vision of this issue concerning this country.

The vaccination campaign in Italy was accompanied by the slogan “Italy is reborn with a flower” and by the symbol of the primrose as a sign of rebirth and hope after the high rate of deaths and the restrictive measures of the lockdown. Various advertisements were broadcast on television to raise public awareness of the importance of vaccination as a means of protection for the individual and for the community, focusing on the emotional aspect of the issue (such as the protection of family members) rather than on the safety of the vaccine [56].

Nevertheless, the website of the Ministry of Health [57], which is updated regularly, provides information about the type of vaccines authorized in Italy, their safety (there is a link to the website of the Italian Medicines Agency, which reports the results of the pharmacovigilance activity using interactive graphs), the possibility of vaccinating particular age groups or immunosuppressed individuals, and the possibility of receiving anti-COVID 19 vaccination at the same time as other vaccinations, as well as a dashboard that collects data and statistics relating to the administration of vaccines throughout the national territory. In addition, there is a section of the site dedicated to the most frequent fake news, with explanations verified by experts from the Ministry of Health and/or the Italian National Institute of Health and is based on scientific evidence, regulations, and national and international documentation [58].

The Italian Pediatric Society conducted a pilot project and enrolled pediatricians as “influencers” to share on their own Facebook profile information from the official page of the scientific society to contrast fake news [59].

However, although for months the media mainly hosted immunologists and medical or scientific experts, the opportunity to take part in the debate was given also to influencers and commentators, resulting in the dissemination of antiscientific opinions, which certainly influenced the diffusion of VH.

Among the determinants of VH most frequently detected by the present study are those closely related to the vaccine, including fear of developing adverse reactions and safety concerns or low confidence in vaccination against COVID-19.

These results are consistent with a survey published in 2016 revealing a high mistrust of the Italian population toward vaccination safety [60].

Indeed, the speed of development and the scarcity of information on vaccines has influenced the reticence of the population and, at the beginning of the vaccination campaign, there was little information about the side effects and safety of the vaccine. Furthermore, the Italian authorities suspended the administration of the Vaxzevria vaccine in March 2021 for a few days after reports of rare side effects related to coagulation disorders. Arguably, this suspension fueled fears of developing adverse vaccine events and, consequently, contributed to the phenomenon of vaccine hesitancy. These findings could be related to another result of the present review, which identifies among the predisposing factors for reluctance the preference to acquire natural immunity rather than through vaccination.

Healthcare professionals and medical students are mainly more concerned about adverse effects than other groups. This finding agrees with previously published studies [61,62,63] and could be related to the refusal of the small number of health professionals to undergo vaccination, which has led the Italian government to issue the decree-law of 1 April 2021, n. 44, establishing COVID-19 mandatory vaccination for Italian healthcare workers until the complete realization of the vaccination plan. Transparent and personalized education of HCWs about the frequency and type of adverse effects related to COVID-19 vaccination could represent a valuable approach to overcoming the fear of this eventuality. Healthcare professionals represent one of the main vehicles of scientifically correct information about vaccinations. Vaccine information obtained from non-healthcare personnel is another study finding influencing vaccine hesitancy. This data is relevant considering that the exponential role of the internet and the mass media in disseminating information has led to the generation of the phenomenon known as “infodemic”. The dissemination of inaccurate information, if not contrasted, can influence the decision-making process of individuals and triggers a self-feeding phenomenon, as people who encounter fake information on social media can, in turn, share it [64].

Among the socio-cultural factors, a low level of education strongly influences the choice to carry out the vaccination against COVID-19, especially among parents. From this point of view, pediatricians need to start orientation courses for parents and pregnant women through adequate counseling and personalized communication strategies to overcome cultural barriers, dialoguing, and providing arguments based on scientific evidence. This need becomes even more contemporary in the light of the recent circular from the Italian Ministry of Health, issued on 9 December 2022, which extends the indication for the use of the Comirnaty (BioNTech/Pfizer) vaccine for the age group between 6 months and 4 years [65].

Therefore, transparent and homogeneous information from HCWs and institutional figures is needed to support preventive behaviours and change personal risk perception.

Regarding political orientation, right-wing beliefs influence the predisposition to vaccination, in agreement with Cadeddu et al. [66], who investigated the confidence of the Italian population in vaccines. This data, however, disagrees with the findings of Engin et al., according to which political conservatism does not impact vaccine beliefs [67].

Among the demographic factors, there seems to be no uniformity between the evidence found in relation to age, while the female gender appears to be a predisposing factor to hesitancy, according to data published by other studies [68].

## 5. Conclusions

Currently, we are facing a gap between the population and science, governments, and institutions [69]. This phenomenon involves both the general population and healthcare professionals, to the point that Italy was the first European country to make vaccination mandatory for this professional category [70].

To heal this breach, it is necessary to strengthen the trust of the population through the implementation of health communication and public education strategies, which cannot prescind from determinants such as competence, correctness, sincerity, faith, consistency, and objectivity [71]. On the other hand, scientific literacy must continue to support families and individuals in discerning evidence from opinions to recognize the real risks and balance them with the benefits [72,73].

Clear communication is the main tool to encourage vaccinations. Since the internet is one of the main sources of interaction, it should be remembered that a lot of information on the web is disseminated by users not having knowledge of the disease or vaccine and promoting fake news circulation.

To counter the infodemic phenomenon, it could be useful for health authorities to implement public platforms (so-called fact-checking) where users can expose and verify doubts about the truthfulness of information. Furthermore, since a low cultural level has proved to be one of the factors predisposing to VH, the information provided by institutional sites must be easy to understand. An Italian study [74] found that Italian regions have websites providing information on COVID-19 vaccines using terminology that is too complex for users with a low level of education to understand. Consequently, the simplification of the texts would allow these population groups to access material about the safety and reliability of vaccines and encourage their propensity to vaccinate.

## 6. Limitations

The studies included in this study are surveys, representing a snapshot of the vaccine hesitancy position in a short time. Furthermore, the results of the studies conducted before and after the start of the vaccination campaign were considered together. Consequently, the incidence of the beginning of the vaccination campaign on the perception of the Italian population was not specifically evaluated, which could be a bias in the data interpretation. Additionally, the surveys used questionnaires with items not always overlapping. Therefore, the results should be interpreted with caution, as the representativeness of the study is not fully guaranteed.

## Figures and Tables

**Table 1:** Summary of the studies included in the present systematic review.

Reference	Target of Population	Period of the Study	Number of Involved Participants	Factors Associated with Vaccination Hesitancy
Miraglia Del Giudice et al. (a) [18]	Pregnant women	September 2021–May 2022	385	Not having a degree; less concern about contracting SARS-CoV-2 infection; information through mass media/internet/social networks
Tomietto et al. (a) [19]	Healthcare professionals and healthcare students	May 2021–June 2021	1226	Worries about unforeseen future effects; being a member of generation X
Peruch et al. [20]	Healthcare workers	From 4 to 31 March 2022	130	Distrust, doubts over safety, and lack of information
Bechini et al. [21]	Healthcare students	February 2021	473	Development of SARS-CoV-2 vaccines and procedures for evaluating clinical trials for marketing authorization too fast to guarantee their efficacy and safety
Raffetti et al. [22]	General population	August 2021	2010	Right and centre-right compared with a left and centre-left political orientation; trust in authorities
Del Riccio et al. (a) [23]	General adult population	From 11 December to 15 December 2020	7563	Using search engines to search for vaccine information; fear of adverse reactions
Savarese et al. [24]	Parents	January–March 2022	1105	Belief that children receive more vaccinations than they should and that it is better to develop immunity rather than be vaccinated; concern that child could have side effects; concern about unsafety; belief that vaccines do not prevent disease; reluctance about pediatric vaccines; distrust in the information received
Lo Moro et al. [25]	Medical students	November 2020–February 2021	902	Survey completion before COVID-19 vaccine authorization; adverse reactions after a vaccination; receiving advice against COVID-19 vaccination from a relative
Lecce et al. [26]	Parents	From 20 September to 17 October 2021.	604	The intention to vaccinate the child was higher in caregivers vaccinated against COVID-19, in those with a bachelor’s degree or higher level of education, and in those with friends/acquaintances who became ill or died due to COVID-19.
Di Giuseppe et al. [27]	Prisoners	From July to October 2021	517	COVID-19 vaccination uptake was significantly higher in females, and in those who reported influenza vaccination uptake, had received information about COVID-19 vaccination from media and newspapers, did not express the need for additional information about COVID-19 vaccine, believed that the COVID-19 vaccine is safe, were involved in working activities in the prison, and had a high school or university degree.
Zarbo et al. [28]	General population	March–May 2021	2015	Lower levels of protective behaviours, trust in institutions and informational sources, frequency of use of informational sources; higher conspirative mentality
Buonsenso et al. [29]	Caregivers of Children with history of SARS-CoV-2 Infection	1 November 2021, and 15 January 2022	123	Occurrence of Long COVID in the child
Moscardino et al. [30]	Young adults (18–40 years)	June 2021	1200	Being aged 30–40 years; residing in northern Italy; having lower educational and income level; being unemployed; not knowing any friends/relatives diagnosed with COVID-19; less social support from friends and family; higher levels of conspiracy theories and negative attitudes toward vaccines; lower levels of attachment to country and perceptions of a just government
Costantino et al. (a) [31]	Pharmacists	From December 2020 to February 2021 and October 2021	2841	The main reasons for changing opinions on vaccination adherence were the introduction of mandatory vaccinations, fear of contracting COVID-19, and limitations on work activities in the case of vaccine refusal.
Miraglia Del Giudice et al. (b). [32]	Parents	From 14 December 2021 to 4 January 2022	430	Respondents who did not receive the COVID-19 vaccine were less educated, with a lower concern about severity of COVID-19, and with a lower perceived risk that their child could be infected by SARS-CoV-2.
Folcarelli et al. [33]	General population who completed primary vaccination series	Between 16 November and 6 December 2021	615	Respondents who self-rated a lower health status after the primary vaccination series, who did not have friends/family members who were diagnosed with COVID-19, who had not received information from official government organizations, and who needed information were hesitant
Genovese et al. [34]	General population	Between 10 February and 12 July 2020	4116	The reasons behind vaccine refusal/indecision were mainly a lack of trust in the vaccine, the fear of side effects, or a lack of perception of susceptibility to the disease.
Colciago et al. [35]	Pregnant women	Between 1 February 2022 and 3 March 2022	538	Having had COVID-19 during pregnancy and having a high-risk perception towards the immunization for the fetus were factors associated with vaccine hesitancy.
Regazzi et al. [36]	Healthcare workers	Between July and November 2021	2142	Increasing age and referring to colleagues to expand knowledge about COVID-19 were positively associated with COVID-19 hesitancy.
Perrone et al. [37]	General population	Between October and November 2021	40	Distrust of the government, infodemic, influence of family, and general anti-vaccine opinions. The results also showed that the most important emotional and cognitive factors associated with hesitancy were anger related to a perceived sense of oppression, emotional avoidance to minimize risk, and anxiety related to potential vaccine side effects
Tomietto et al. (b) [38]	Nurses	From May to June 2021	430	Low mistrust about the vaccine’s benefit, concernsabout commercial profiteering, preference for natural immunity, unexpected future effects
Monami et al. [39]	Physicians	1 to 28 January 2021	7881	The vaccine hesitancy rate was correlated with prior SARS-CoV-2 infection, diabetes, adverse events at previous vaccinations, and refusal of 2020 flu vaccine, and was mainly motivated by concerns about vaccine adverse events.
Papini et al. [40]	Healthcare Workers	From 19 February to 23 April 2021	2137	Female sex, a lower education level, greater hesitancy, and refusal to adhere to flu vaccination campaigns were predictors influencing the aversion to mandatory vaccination.
Bianco et al. [41]	Parents	Between April and May 2021	394	Respondents who had not graduated, those who did not believe that this vaccination was useful, those who did not receive this vaccine, those who did not obtain information from physicians, and those who needed additional information were more likely to be highly hesitant.
Contoli et al. [42]	Elderly	From January 2020 to December 2020	1876	Not having received vaccination against influenza during the previous flu season, lower risk of having had a death from COVID-19 among family or friends. The hesitancy group was significantly more likely to be worried and they did not know if consequences of the disease would be serious for them.
Costantino et al. (b) [43]	Liver transplant recipients	February 2021	190	The fear of adverse effects was the main reason for refusal.
Zona et al. [44]	Parents	From 15 July to 16 August 2021	1799	Younger than 40 years of age, with a secondary-school or three-year degree, freelance, with a family income below €28,000, with an erroneous perception of the risk of COVID-19 as a disease and with fear of anti-COVID vaccination
Blanchi et al. [45]	Patients on dialysis	March 2021	Not deducible	Concerns about side effects and vaccine efficacy
Scoccimarro et al. [46]	Diabetic patients	From 1 January to 30 April 2021	502	Lower adherence to medical prescriptions and/or reduced concerns over their health
Salerno et al. [47]	Students	From 7 May to 31 May 2021	2667	Statistically significant higher VH and vaccine resistance rates were found for viral vector than mRNA COVID-19 vaccines.
Del Riccio et al. (b) [48]	General population	From 11 December to 15 December 2020	7605	Lower level of trust in institutions, being hesitant about other vaccinations, and being employed
Costantino et al. (c) [49]	Celiac disease patients	Between 22 February and 26 February 2021	103	The main reasons for hesitancy were fear of adverse events and/or distrust of the fast vaccine production, not being afraid of COVID-19, thinking the vaccine would not be efficient in protecting against disease, and the decision being influenced by celiac disease.
Montalti et al. [50]	Parents	Between December 2020 and January 2021	4993	Female parents/guardians of children aged 6–10 years, ≤29 years old, with a low educational level, relying on information found on the web/social media, and disliking mandatory vaccination policies
Reno et al. [51]	General population	January 2021	1011	Past vaccination refusal, ages between 35 and 54 years, female gender, low educational level, low income, absence of comorbidities, safety and efficacy of the vaccine
Di Gennaro et al. [52]	Healthcare Workers	From 1 October to 1 November 2020	1723	Using Facebook as the main information source, being a non-physician HCW, lack of trust in vaccine safety, and receiving little or conflicting information about vaccines
Fedele et al. [53]	Parents	Between November 14 and 28, 2020	640	Vaccine refusal was attributed to safety concerns in 76% of parents. Specific vaccine attributes further reduced the acceptance rate. Female gender, a younger age, and a lower education level were associated with non-adherence to vaccination.

**Table 2:** Most frequently encountered factors predisposing vaccine hesitancy in the Italian population.

Predisposing Factor	Number of Involved Studies	Pregnant Women/Parents	Medical Students/Health Professionals	Vulnerable People	General Population
Low level of education	11	7	1	1	2
Information through mass media/internet	6	2	1	1	2
Lack of information	4	1	2		1
Information obtained did not come from physicians	4	1	2	-	1
Distrust in authorities	5	-	-	-	5
Fear of adverse reactions/long COVID	12	2	4	3	3
High conspirative mentality	2	-	-	-	2
Preference for natural immunity/having had COVID-19 before the vaccination	4	2	2	-	-
Refusal of vaccinations/low level of protective behaviour	11	2	2	2	5
Female gender	5	2	1	1	1
Less concerned about contracting SARS-CoV-2 infection	9	3	1	3	2
Doubts over safety/distrust in COVID-19 vaccination	11	3	4	2	2
Friends/family members diagnosed with COVID-19	4	1	-	1	2
Efficacy of the vaccine	5	2	-	2	1

## Data Availability

Data supporting this systematic review are available in the reference section.
